# Factors Influencing Serum Vitamin D Concentration in Turkish Children Residing in İzmir: A Single-Center Experience

**DOI:** 10.4274/jcrpe.1938

**Published:** 2015-12-03

**Authors:** Pamir Gülez, Hüseyin Anıl Korkmaz, Dilek Özkök, Demet Can, Behzat Özkan

**Affiliations:** 1 Dr. Behçet Uz Children Disease and Surgery Training and Research Hospital, Clinic of Pediatrics, İzmir, Turkey; 2 Dr. Behçet Uz Children Disease and Surgery Training and Research Hospital, Clinic of Pediatric Endocrinology, İzmir, Turkey; 3 Dr. Behçet Uz Children Disease and Surgery Training and Research Hospital, Clinic of Pediatric Allergy and Immunology, İzmir, Turkey

**Keywords:** risk factors, 25-hydroxyvitamin D, Vitamin D, infant

## Abstract

**Objective:**

The aim of this study was to examine the vitamin D status of children and to determine the factors influencing serum 25-hydroxyvitamin D [25(OH)D] concentration in Turkish infants living in İzmir.

**Methods:**

In this study, we examined the serum 25(OH)D levels of 100 infants aged 1 to 24 months and of 22 mothers from İzmir, Turkey. The study also included a questionnaire given to the mothers to acquire data on the demographic characteristics of the infants and their mothers as well as information on vitamin D supplementation, clothing habits, and sunlight exposure.

**Results:**

Vitamin D deficiency was present in 31% of infants and 81.8% of mothers. Twenty-four male (42.9%) and 7 female (15.9%) infants were found to be vitamin D deficient (<20 mg/dL); 9 male (16.1%) and 17 female (38.6%) infants to be vitamin D insufficient (20-30 mg/dL); and 23 male (41.1%) and 20 female (45.5%) infants were vitamin D sufficient (>30 mg/dL). Only 63% of the infants were receiving vitamin D supplementation and 52% were said to be having regular exposure to sunlight. Mean serum vitamin D levels were lower in infants whose mothers were dressed according to the culture of traditional covered clothing (44%) compared to those infants whose mothers’ dressing style provided more exposure to sunlight.

**Conclusion:**

We conclude that low exposure to sunlight, inadequate use of vitamin D supplementation, and large family size are factors influencing the vitamin D status of Turkish children living in the inner city of İzmir.

**WHAT IS ALREADY KNOWN ON THIS TOPIC?**

25-hydroxyvitamin D [25(OH)D] is the major circulating form of vitamin D, and its concentrations are the best available indicator of total body vitamin D status. Serum 25(OH)D level depends on factors such as sunlight exposure, seasonal variation, latitude over 40˚ north or south, skin pigmentation, clothing habits, age, maternal vitamin D stores and exclusive breastfeeding, malabsorption disorders, chronic diseases, medications, as well as dietary vitamin D intake or vitamin D supplementation.

**WHAT THIS STUDY ADDS?**

Vitamin D status of inner-city children aged 1-24 months who reside in İzmir and the factors related with low vitamin D status.

## INTRODUCTION

25-hydroxyvitamin D [25(OH)D] is the major circulating form of vitamin D and its blood level is the best available indicator of total body vitamin D status. Serum 25(OH)D concentration depends on factors such as sunlight exposure, seasonal variation, latitude over 40˚ north or south, skin pigmentation, clothing habits, age, maternal vitamin D stores and exclusive breastfeeding, malabsorption disorders, chronic diseases, medications, dietary vitamin D intake or vitamin D supplementation (1,2,3,4).

Vitamin D insufficiency and nutritional rickets was an important health problem in Turkey until recent years (5), but the incidence is now much lower, following the nationwide vitamin D prophylaxis programme undertaken by the Turkish Ministry of Health in 2005. This programme provides free vitamin D drops containing 400 IU daily for all children under 12 months of age (6).

To date, very few studies on prevalence of vitamin D status of inner city Turkish children under 2 years of age have been undertaken (5,7,8,9,10,11). İzmir province is located on latitude 38.25˚ N, with a sunny climate from April through November. In addition, air pollution is minimal in this geographical area. Thus, the environmental factors allow sufficient sunlight to maintain adequate vitamin D status by dermal synthesis. On the other hand, the incidence of vitamin D insufficiency has been reported to be high in infants living in İzmir (9).

The aim of this present study was to determine the vitamin D status of inner city children aged 1 to 24 months residing in İzmir and to investigate the factors related with low vitamin D status.

## METHODS

This study was conducted between July and September 2012 at Dr. Behçet Uz Children’s Hospital, Clinic of Pediatrics, İzmir. White Caucasian neonates born after 36 weeks of gestational age whose mothers were resident of İzmir were included in the study. This research was undertaken on random selection of 100 infants (44 females) between 1 and 24 months of age who presented to our outpatient clinic for health checkup. Conditions such as sepsis, malabsorption disorders, congenital or acquired bone disease, chronic diseases, use of long-term medications especially affecting vitamin D metabolism such as anticonvulsants and glucocorticoids and use of sunscreen creams constituted the criteria for exclusion from the study.

At presentation, data on demographic details, clinical and laboratory parameters were collected. Ethical approval for this study was obtained from Dr. Behçet Uz Children’s Hospital Ethics Committee. Informed consent was taken from the mothers to recruit the mother and her child for the study.

Mothers were asked individually by a single researcher to answer an oral questionnaire that contained sections about demographic characteristics and vitamin D supplementation for children and their mothers. The questionnaire included questions on mother’s age, educational and employment status, clothing habits (covered or traditional dressing style was defined as wearing clothing covering head, arms and legs but not hands and face, whereas uncovered dressing style was defined as head and arms uncovered), as well as vitamin D (multivitamin supplementation of mothers in pregnancy with recommended minimum standard dose, 400 IU vitamin D) and calcium supplementation in women with low calcium intake during pregnancy. Educational status of each mother was grouped according to the duration of education as: 0-8 years graded as illiterate or primary school graduate and >9 years graded as high school or university graduate.

The questions on the offspring consisted of age, gender, duration of breastfeeding, vitamin D supplementation and compliance, the dose of the vitamin D supplement (Devit-3 oral solution, Deva Drug Factory, 4. Levent, İstanbul, Turkey, contains 133 IU vitamin D in 1 drop), sunlight exposure of areas of body parts and duration time, and number of siblings of the index child. We categorized daily intake of 400 or 800 IU vitamin D based on compliance. Vitamin D supplementation of 400 or 800 IU vitamin D every day, from birth to 12 months of age, was considered as good compliance, while intake of less than seven days or discontinuation before 12 months of age was considered as poor or inadequate compliance. Regular sunlight exposure was defined as exposure to sunshine of the children and mothers for a duration of at least 30 minutes/day for more than 4 days of a week. Serum 25(OH)D levels of all children and the mothers whose children were less than 6 months at the time of study enrollment were measured. However, 13 mothers (7 breastfeeding) did not consent for their bloods to be collected.

Plasma samples were obtained in EDTA tubes, were mixed, and centrifuged for 15 minutes at 2500 rpm. The sera were stored at -20 °C until the day of analysis. Serum calcium, phosphorus, and alkaline phosphatase (ALP) were measured spectrophotometrically. Total 25(OH)D levels in the sera were assayed using electro-chemiluminescence immunoassay (ECLIA) method (Cobas e601 autoanalyser, Roche Diagnostic GmbH, Mannheim, Germany). The manufacturer gave the analytical measuring range as 3.00 to 70.0 ng/mL. Values below the limit of detection are reported as <3.00 ng/mL, and values above the measuring range are reported as >70.0 ng/mL. The intra-assay precision was 2.2% to 6.8%, and the inter-assay precision was 3.4% to 13%. Concentration of 25(OH)D for categorizing vitamin D status were: sufficiency> 30 ng/mL, insufficiency 20-29 ng/mL, and deficiency <20 ng/mL (12,13). For statistical evaluation, the children were divided into two groups according to their serum vitamin D levels as <20 ng/mL and >20 ng/mL because the 2010 Institute of Medicine report (13) set the lowest normal value and the definition of deficiency at 20 ng/mL. The infants were also divided into three groups by age as 1 to 6 months, 7 to 12 months, and 13 to 24 months.

Statistical analysis was performed using the SPSS for Windows (version 17.0). Student t-test, one-way ANOVA, post hoc tests, cross tabs, and chi-square tests were used for multiple correlation analysis. A multiple linear regression method was used to predict serum 25(OH)D levels from potential independent variables. A p-value of <0.05 was considered statistically significant.

## RESULTS

**Figure 1:**
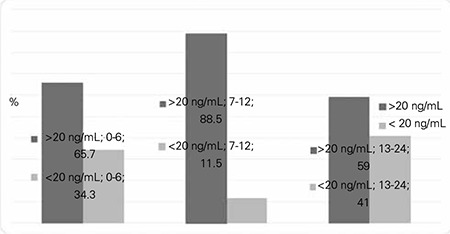
Serum 25-hydroxyvitamin D status by age groups

The age of the 100 infants ranged from 1 to 24 months, and the mean age was 10.4±6.7 months. Tables 1a and 1b demonstrate the demographic characteristics of children and mothers, respectively. In the infants, the mean concentration of serum 25(OH)D was 28.1±14.7 ng/mL (range 3.0-70 ng/mL), while that of the mothers was 10.3±8.8 ng/mL (range 2.9 to 29.6 ng/mL). Serum calcium concentration was 10±0.5 [mean ± standard deviation (SD)] mg/dL, serum phosphorus concentration was 5.9±0.8 mg/dL (mean ± SD), and serum ALP concentration was 195.5±79.9 IU/L (mean ± SD) (Table 1b). Thirty-one percent of the infants were vitamin D deficient, 26% were vitamin D insufficient, and 43% were sufficient (Figure 1). Eighty-eight infants who received 400 IU of vitamin D supplementation had a serum 25(OH)D concentration of 27.4±13.8 ng/mL (mean ± SD), while 10 infants who received 800 IU vitamin D supplementation had a 25(OH)D concentration of 36.2±19.8 ng/mL (mean ± SD). In infants who received 400 IU of vitamin D supplementation, 36 (40.9%) were sufficient, 25 (28.4%) infants were insufficient, and 27 (30.7%) infants were deficient. In infants who received 800 IU vitamin D supplementation, 7 (70%) were sufficient and 3 (30%) infants were deficient. Mean serum 25(OH)D concentration of infants aged 1-6 months of mothers with traditional covered clothing (20.7±12.6 ng/mL) was significantly lower than the mean serum 25(OH)D concentration of infants aged 1-6 months of mothers with uncovered clothing (35.5±11.3 ng/mL) (p=0.001). In this age group, the mean serum 25(OH)D concentration of the children of mothers who had received vitamin D supplementation (29.1±14.5 ng/mL) was higher than the mean serum 25(OH)D concentration of the infants of mothers who had not received vitamin D supplementation (24.9±12.8 ng/mL) (p=0.439). The serum 25(OH)D concentration of 29 exclusively breastfed infants was 23.3±12.8 ng/mL (mean ± SD) with a range of 3.0-50.8 ng/mL, while serum 25(OH)D of their mothers was 11.5±9.6 ng/mL (mean ± SD) with a range of 3.0-29.58 ng/mL.

Clinical and laboratory characteristics of the mothers of infants are shown in Table 2a and maternal data are shown in Table 2b. All mothers recruited in this study were vitamin D insufficient. The breastfeeding period ranged between 1 and 8 months with mean ± SD of 3.4±1.9 months. All of these mothers were unemployed, 12 had received vitamin D and 11 calcium supplementation during their pregnancies. Nine of them had regular sunlight exposure, 13 had traditional dressing, and 4 had more than 3 children. Among 29 exclusively breastfed infants, 28 were given vitamin D drops, the vitamin D drops were given regularly to 19 infants, and 21 infants were exposed to sunlight regularly.

Mean serum 25(OH)D concentration of infants of mothers with traditional covered clothing was 24.5±15.3 ng/mL and was significantly lower than the mean serum 25(OH)D concentration of infants of mothers with uncovered clothing (32.2±12.8 ng/mL) (p=0.008). In mothers with uncovered clothing, 25 (54.3%) infants were sufficient (mean 25(OH)D concentration: 41.4±8.7 ng/mL), 15 (32.6%) infants were insufficient (mean 25(OH)D concentration: 25.2±2.0 ng/mL), and 6 (13%) infants were deficient (mean 25(OH)D concentration: 11.5±3.9 ng/mL). In mothers with traditional dressing, 18 (33.3%) infants were sufficient (mean 25(OH)D concentration: 41.6±11.7 ng/mL), 11 (20.4%) infants were insufficient (mean 25(OH)D concentration: 25.1±2.6 ng/mL), and 25 (46.3%) infants were deficient (mean 25(OH)D concentration: 11.8±5.5 ng/mL). The educational status of the mothers, vitamin D and calcium supplementation during pregnancy, frequency and duration of exposure to sunlight, regular vitamin D supplementation of the infants did not significantly affect the serum 25(OH)D concentration in the infants.

Vitamin D deficiency was significantly higher in males (n=24) than females (n=7) (p=0.005). Serum 25(OH)D concentration in infants 1-6 months old were found to be higher in infants who received vitamin D drops (28.9±14.5 ng/mL) compared to those who were not supplemented with vitamin D drops (16.7±13.1 ng/mL) (p=0.015). In infants 13-24 months of age, the serum 25(OH)D concentration (11.4±1.8 ng/mL) was significantly lower than that of infants 1-12 months of age (15.5±1.9 ng/mL) (p=0.003). Mean serum 25(OH)D levels of infants with 1 or 2 siblings was found to be higher (29.7±15.1 ng/mL) than those who had more than 2 siblings (17.5±14.4 ng/mL) (p=0.017). Serum 25(OH)D was also higher in infants regularly exposed to sunlight (32.7±11.9 ng/mL) compared to those intermittently exposed to sunlight (13.6±11.9 ng/mL) (p=0.023). The difference of serum 25(OH)D concentration in infants who received 400 IU (27.4±13.8 ng/mL) and 800 IU (29.1±18.5 ng/mL) daily was not found to be statistically significant (p=0.846).

In multiple regression analysis, regular vitamin D supplementation and maternal education were independent of serum 25(OH)D concentration. A positive correlation was found between sunlight exposure during pregnancy and serum 25(OH)D concentration of the offspring. In infants aged 1-6 months, a negative correlation was found between the number of siblings and 25(OH)D levels (Table 3).

## DISCUSSION

The Turkish Ministry of Health provides a daily dose of 400 IU of vitamin D supplementation to infants during the first year of life and to all pregnant women visiting primary care clinics for routine pregnancy follow-up examinations (6). Previous studies have reported a prevalence of vitamin D deficiency in Turkish infants as 8-12% (14,15) in groups similar to those of our study. In our study, which was conducted on a small but representative sample of İzmir inner city population, the prevalence of vitamin D deficiency was 57% in the infants aged 1 to 24 months and 81.8% in their mothers, figures which indicate a higher prevalence in İzmir, which has an abundance of sunny days throughout the year. Higher rates of maternal vitamin D deficiency predispose the infants to the risk of rickets during early infancy. Several studies from Turkey have shown that vitamin D insufficiency or deficiency were prevalent among the mothers who did not have a lifestyle that would expose them to sunshine. Low sunshine exposure due to spending more time indoors and covered clothing habits, low intake of vitamin D, and low educational level predispose mothers and their breastfed infants to vitamin D deficiency (11,16,17,18,19). We found that the duration of sun exposure of mothers significantly correlated with maternal vitamin D status. Our results also demonstrate that the serum 25(OH)D concentrations of the mothers depend on their clothing habits and that covered traditional clothing predisposes to vitamin D deficiency. Maternal vitamin D status influences the vitamin D status of exclusively breastfed infants during the first 2 months of life, but thereafter, infant vitamin D status is more directly affected by sunlight exposure and vitamin D supplementation (1,2,3). In a similar line, we found that the vitamin D status of breastfed infants correlated with maternal vitamin D status and that most breastfed infants and their mothers were vitamin D sufficient.

Similar results have been reported by others (10,16,20,21). We also found that vitamin D insufficiency was more frequent in infants 13 to 24 months old in comparison to those in their first year of life. This result could be explained by the fact that the Turkish Ministry of Health provides free daily vitamin D supplements only for infants in their first year of life. Thus, this finding raises an important argument for provision of free vitamin D supplementation also for infants beyond 1 year of age.

In our study group, vitamin D supplementation in a dose of 400 IU/day was found to be adequate in maintaining the serum 25(OH)D at optimal concentration, a finding which has also been reported in previous studies (18,22). However, since the blood samples in our study were collected during summer months, a higher supplementary dose may be required during the winter.

Korchia et al, (20) in their study from Israel, found a significant negative relationship between 25(OH)D concentration and number of siblings of the index child. Our findings are similar and the only possible explanation we can suggest for this finding would be that larger families were less conscious of the benefits of vitamin D supplementation and did not benefit from the free provision of this supplement for pregnant women and infants.

Calcium and phosphorus are absorbed in the upper small bowel by an active transcellular transport mechanism stimulated by 1,25-dihydroxyvitamin D. During the phases of rapid growth during infancy and early childhood, calcium and phosphorus absorption is very important to allow for bone mineralization. Although İzmir has an abundance of sunshine almost throughout the year, we found a high prevalence of vitamin D insufficiency in infants. This finding shows the importance of checking the 25(OH)D concentration of infants and mothers and to provide treatment or supplementation if necessary. In addition, the adequacy of dietary calcium intake needs to be optimize the bone health during the rapid growing phase of infancy and early childhood. Rickets may also arise from decreased mineralization of the bone matrix due to deficiencies of calcium or phosphate. We observed no patient with signs of rickets in our study, because dietary calcium intake was adequate in our patients despite vitamin D deficiency or insufficiency.

Our results do not reflect the entire population of Turkey as İzmir has a temperate climate with clear skies throughout the year providing opportunity for abundant exposure to sunlight for vitamin D production. More studies covering other regions of Turkey and encompassing populations with different cultural practices are required to identify the burden of vitamin D deficiency in Turkish children.

To conclude, our results demonstrate that vitamin D deficiency is common in the inner city population of İzmir. Even though vitamin D supplementation is universally available for infants, further efforts are required to educate the population about the benefits of supplementation. A concerted effort is required from the Ministry of Health and health professionals in achieving this goal.

## Figures and Tables

**Table 1a:**
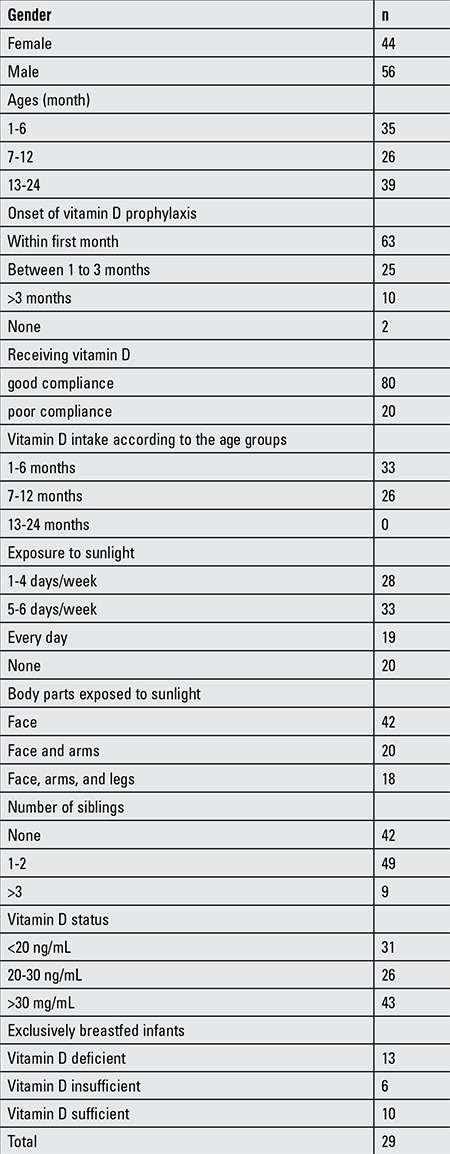
Clinical and laboratory characteristics of study groups

**Table 1b:**
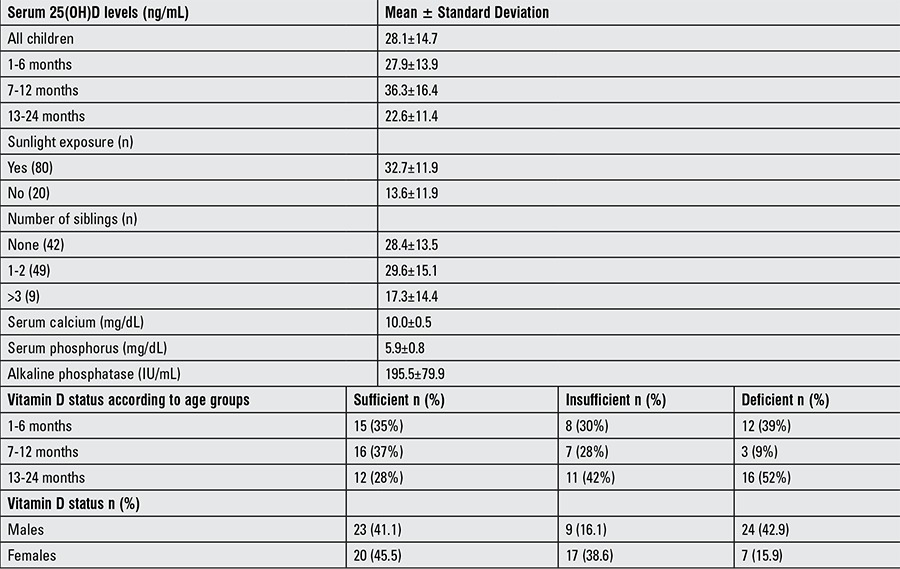
Biochemical results of the study group

**Table 2a:**
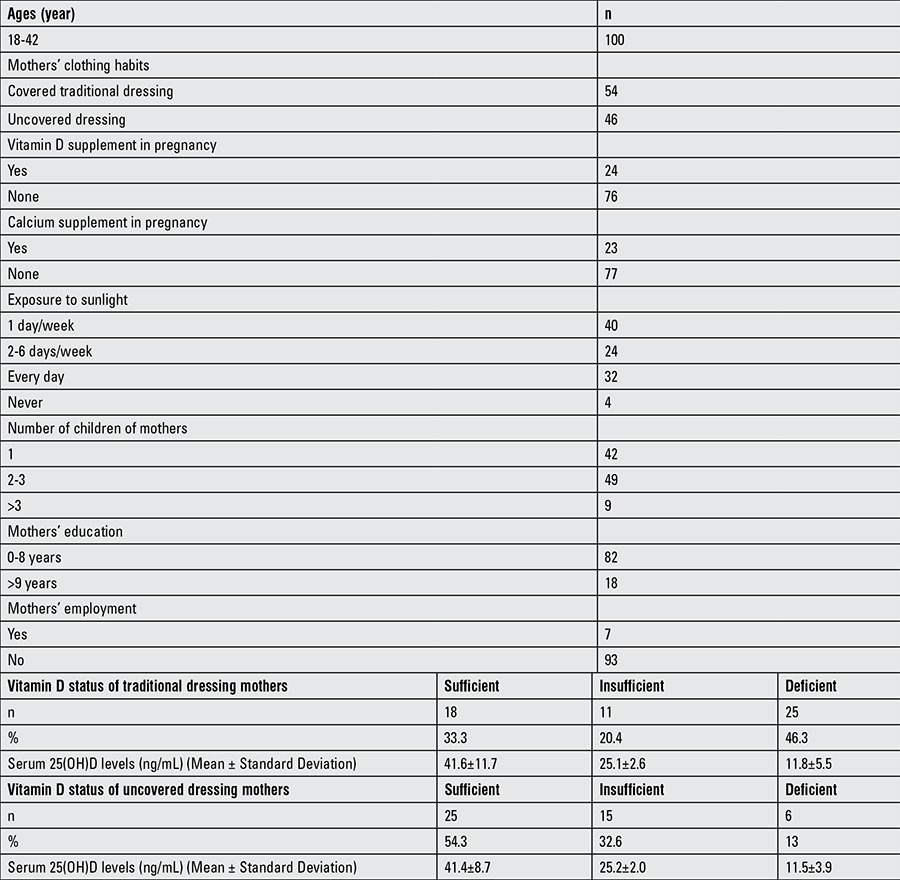
Clinical and laboratory characteristics of the mothers of infants

**Table 2b:**
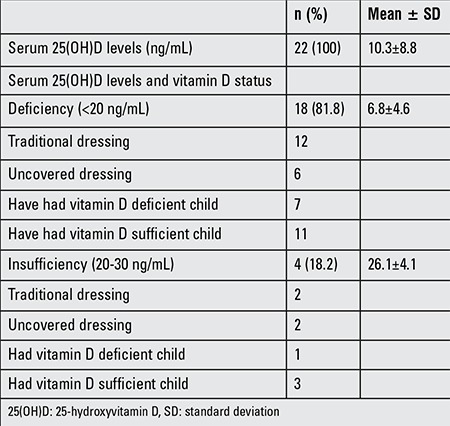
Clinical results of the mothers and associated factors

**Table 3:**
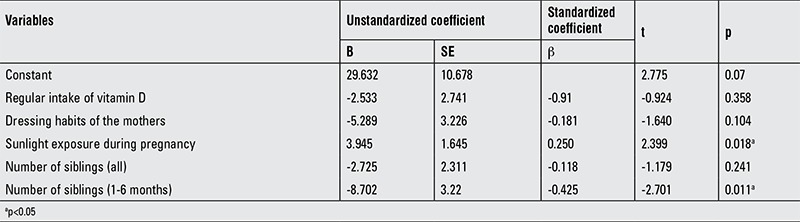
Multiple linear regression analysis of factors associated with serum 25(OH)D levels of children (n=100)
